# Decidualized Ovarian Mass Mimicking Malignancy

**DOI:** 10.1155/2015/217367

**Published:** 2015-04-15

**Authors:** Lufee Wong, Vola Botolahy, Thibault Carteret, Marion Marty, Jean-Luc Brun

**Affiliations:** ^1^Department of Obstetrics and Gynecology, University Hospital Pellegrin, 33076 Bordeaux, France; ^2^Department of Radiology, University Hospital Saint-Andre, 33000 Bordeaux, France; ^3^Department of Pathology, University Hospital Haut-Leveque, 33604 Pessac, France

## Abstract

Deciduosis classically occurs in the context of known endometriosis in the pelvis, most commonly in the ovaries, but also in the peritoneum. However, ovarian deciduosis outside the context of endometriosis is rare and makes diagnosis difficult, especially as the sonographic appearance suggests a malignant process. We report a case of decidualized ovarian mass in a patient without prior history of endometriosis that mimicked an ovarian malignancy. MRI may be a useful imaging modality to monitor these lesions and guide management. Consultation with a multidisciplinary team accustomed to such conditions will help to tailor the management to each individual.

## 1. Introduction

Decidualization refers to the process in pregnancy whereby the normal endometrium of the uterus undergoes specialized transformation to support the growing fetus. This is largely a hormonal response to progesterone. Ectopic deciduosis is classically related to a context of known endometriosis [1–4]. It occurs in the pelvis, most commonly in the ovaries, the anterior and posterior surfaces of the uterus, and the omentum but also in the peritoneum, pleura, and bladder. It is benign in nature and undergoes spontaneous regression in the postpartum period [1]. However, ectopic decidua can grow during pregnancy to resemble a malignant tumor, particularly when the structural changes present as sinister sonographic features. The diagnostic dilemma is to differentiate benign decidualized tissue that can be managed conservatively from malignant tumor that will require extensive invasive procedures and potentially early termination of the pregnancy. We report a case of decidualized ovarian mass in a patient without any previous history of endometriosis that strongly mimicked an ovarian malignancy that resulted in a premature delivery.

## 2. Case Report

**Figure 1:**
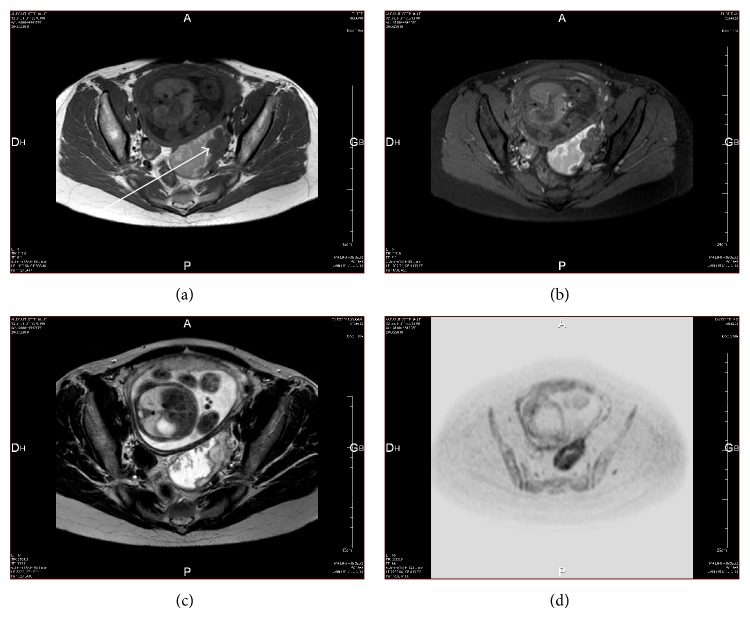
Magnetic Resonance Imaging features of the left decidualized ovarian mass. Axial T1 weighted image (a) demonstrates an intermediate signal intensity of the solid component in the left ovary (arrow), which remains unchanged on fat suppressed T1 weighted image (b). The solid component exhibits an intermediate T2 weighted signal (c) and a high diffusion-weighted signal (d).

A 32-year-old primigravida presented at 21 weeks of pregnancy for investigation of left pelvic pain. She had no prior history of endometriosis. An early transvaginal ultrasound in first trimester did not report any adnexal masses. Transvaginal ultrasound revealed a large 9 cm left ovarian mass that was multiloculated with nodule thickening of septas and of the wall with a positive Doppler flow inside. The CA 125 assay was 50 IU/L. A diagnostic laparoscopy was proposed to the patient due to the concern of possible borderline or even malignant tumor, but she declined this procedure. At 24 weeks, a Magnetic Resonance Imaging (MRI) without contrast was performed as the patient refused gadolinium injection. The heterogeneous mass had not increased in size (7.7 cm). The solid component exhibited an intermediate T2 weighted signal and a high diffusion-weighted imaging (DWI) signal, suggesting the possibility of a borderline component (Figure 1). There was no ascites, peritoneal carcinomatosis, lymphadenopathy, nor any signs of compression. CA 125 and MRI remained unchanged at 28 weeks.

Given the absence of symptomatology and the stable imaging, the pregnancy was prolonged until an acceptable level of prematurity, as recommended by the French national multidisciplinary network for the management of pregnancy-associated cancers. Corticosteroids for accelerating fetal lung maturation were administrated at 29 and 33 weeks in case of acute complications requiring immediate preterm birth. The follow-up of pregnancy was uneventful until 35 weeks.

A planned caesarean section was performed at 35 weeks and this resulted in the birth of a healthy baby girl weighing 2100 g, with Apgar 10 at 1 and 5 minutes. At laparotomy, the left ovarian tumor measured 9 cm in diameter and had an irregular white surface covered by red punctations. Focal zones of the cortical surface had partially ruptured and small cysts containing clear yellow fluid were observed under the cortex of the ovarian tumor. The peritoneal surfaces of the uterus, the bowels, and the pouch of Douglas were also covered by reddish punctations and flames. The right ovary was 3 cm in diameter and carried a small simple cyst of 2 cm. A left salpingo-oophorectomy was performed. Multiple peritoneal biopsies were carried out, as well as the removal of the right ovarian cyst. Her postoperative course was unremarkable.

**Figure 2:**
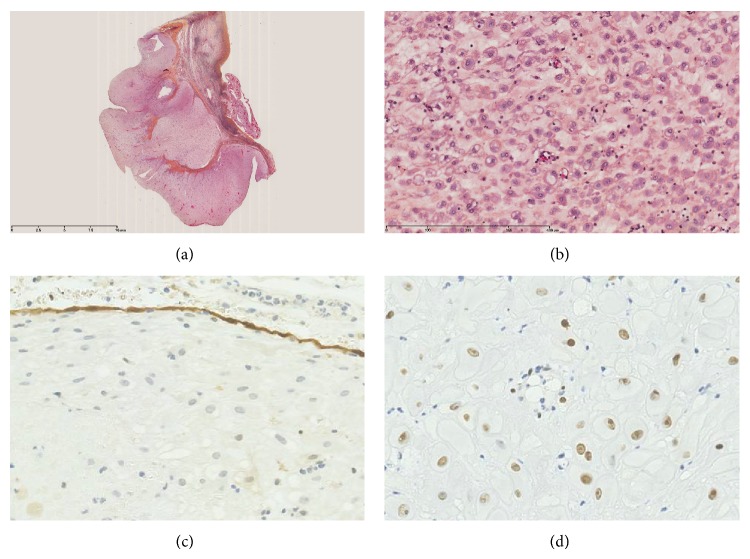
Microscopic examination of the left decidualized ovarian mass. Low magnification (a) shows an exophytic lesion developed on ovary surface (hematin eosin, ×0.5). High magnification (b) shows bland eosinophilic and granular epithelioid cells with no atypia nor mitotic activity (hematin eosin, ×20). By immunohistochemistry with calretinin (c) the epithelioid cells are negative (positive mesothelial cells are seen at the top), whereas they are stained by anti-progesterone receptor antibody (d) (brown nuclear positivity).

Macroscopic examination of the left adnexal mass revealed a mixed ovarian tumor with solid and cystic components and vegetations inside and outside the cyst measuring up to 5 mm in height. On sectioning, pale yellow fluid was present. Frozen section showed a nonepithelial lesion, with no evidence of malignancy, and the overall appearance resembled a functional ovarian cyst. The final microscopic histological examination of the left ovarian tumor showed a lesion consisting of luteinized cells, with a large eosinophilic granular cytoplasm and rounded nucleus cells, sitting in an edematous stroma (Figure 2). The papillary excrescences consisted only of dense fibrous tissue. There was no sign of endometriosis nor malignancy. Similar lesions were found on the peritoneal biopsies, the right ovarian cyst, and the peritoneal cytology. Immunohistochemical analysis showed strong positivity with progesterone receptors and negativity for mesothelial markers (calretinin and WT1). These findings supported the diagnosis of peritoneal and ovarian deciduosis.

## 3. Discussion

Isolated ovarian deciduosis in the absence of active endometriosis or previous history of endometriosis is rare. To our knowledge, only one case has been described in the literature previously [1]. However, the diagnosis was incidentally made during an uncomplicated elective caesarean section at term. A right-sided ovarian mass of 7 cm in diameter demonstrated a hyperneovascularized appearance over the external surface suspicious of malignancy. Hemorrhagic nodules within the omentum also suggested metastases. On close histopathological examination, no borderline changes, atypia, or malignancy was observed and immunohistochemistry supported the diagnosis of ovarian deciduosis. Likewise in our patient, the complex ovarian mass mimicked malignancy, but unlike the previously published case report, she presented with clinical symptoms in second trimester. Indeed, ovarian deciduosis is often asymptomatic and the diagnosis is mainly incidental at conventional obstetric ultrasound or during cesarean section. However, clinical manifestations, such as abdominal pain, as occurred in our patient, and dystocia, have been described [1]. Should the patient become symptomatic, most presentations typically occur in the second or third trimester with minimal or no symptoms detectable in the first trimester and the patients usually have a normal first trimester ultrasound examination. Upon discovery of the ovarian mass, the volume generally remains grossly stable throughout the rest of the pregnancy, as was the case in our patient [4–6].

Another aspect of ovarian deciduosis that often complicates its diagnosis is the formation of a complex ovarian mass that bears sonographic features mimicking a malignant ovarian tumor. At ultrasound, decidualization typically appears as a mixed solid-cystic tumor with rapidly growing intracystic vegetations, richly vascularized on color Doppler [3, 4]. These sonographic appearances pose a diagnostic dilemma particularly if typical features of an endometrioma, such as fine internal echoes (the “ground glass” appearance), are not observed [3–5]. In addition, color Doppler examination is not useful in differentiating benign endometrioma from malignancy during pregnancy since low resistance flow may be present in both conditions [3, 5]. Other features that may be present include irregular internal walls, although certain features of malignancy such as ascites are notably consistently absent [3, 4]. It is worthy to note, however, that true ovarian malignancies complicating pregnancies are extremely uncommon [7]. Given this challenge in achieving a definitive diagnosis upon discovery of the ovarian mass, expectant management over four to six weeks in the first instance is not an unreasonable approach to evaluate any concerning trends in the sonographic features and/or CA 125 levels, so as to help distinguish between benign and malignant ovarian masses.

MRI may be useful in distinguishing benign ovarian masses from malignant masses. Malignancy is suggested when the solid component demonstrates a strong and early gadolinium enhancement on perfusion weighted imaging (PWI), high DWI signal, and an intermediate T2 weighted signal [8]. In our case, the use of PWI with gadolinium injection may have been helpful to differentiate the enhancing high-signal intensity of intracystic malignant growths from the nonenhancing benign vegetations [3, 8]. However, while no damage to a developing human fetus caused by gadolinium exposure has been documented, caution is advised [9]. In our case, the presence of the two latter features (i.e., high DWI signal and T2 weighted isosignal) did not allow the exclusion of malignancy and thus mandated close regular surveillance with both regular ultrasound monitoring and MRI. The stability of the ovarian mass over time on both imaging modalities, however, is reassuring and helped to prolong the pregnancy and delay surgery [10]. Tumor markers, such as CA 125, CA 19-9, and alpha-fetoprotein, have limited use in the evaluation of pelvic masses since they are physiologically elevated during pregnancy. In spite of this, a longitudinal trend of stable, mildly elevated values may render a malignancy process less likely.

From the pathological point of view, the macroscopic appearance of peritoneal lesions can be mistaken for malignant mesothelioma, tuberculosis, or peritoneal carcinomatosis [11]. The bland cytologic aspects (cells without any atypia) and immunohistochemistry give the final benign diagnosis: decidual cells are hormone receptors positive and do not show positivity with epithelial (cytokeratin) nor mesothelial (calretinin, WT1) markers, confirming the diagnosis of ovarian deciduosis [11].

Given this malignancy-mimicking feature of deciduosis, the prenatal management of these lesions is challenging. Given the stable clinical picture, imaging results, and the infrequent risk of torsion beyond 20 weeks of pregnancy, expectant management was thought to be a reasonable approach until the risks of prematurity were more acceptable. The decision to terminate the pregnancy by elective cesarean section at 35 weeks was a compromise solution between the risk of malignancy and fetal prematurity.
